# Genomic signature of *MTOR* could be an immunogenicity marker in human colorectal cancer

**DOI:** 10.1186/s12885-022-09901-w

**Published:** 2022-07-26

**Authors:** Chenxing Wang, Batuer Aikemu, Yanfei Shao, Sen Zhang, Guang Yang, Hiju Hong, Ling Huang, Hongtao Jia, Xiao Yang, Minhua Zheng, Jing Sun, Jianwen Li

**Affiliations:** 1 Department of General Surgery, Ruijin Hospital, Shanghai Jiao Tong University School of Medicine, Shanghai, 200025 China; 2 Shanghai Minimally Invasive Surgery Center, Ruijin Hospital, Shanghai Jiao Tong University School of Medicine, Shanghai, 200025 China

**Keywords:** *MTOR*, Colorectal cancer, Immune infiltrates, Microsatellite instability, Whole-exome sequencing

## Abstract

**Background:**

The mTOR signaling pathway plays an important role in cancer. As a master regulator, the status of *MTOR* affects pathway activity and the efficacy of mTOR inhibitor therapy. However, little research has been performed to explore *MTOR* in colorectal cancer (CRC).

**Methods:**

In this study, gene expression and clinical data were analyzed using The Cancer Genome Atlas (TCGA) and the Gene Expression Omnibus (GEO) databases. Signaling pathways related to *MTOR* in CRC were identified by Gene Ontology (GO), Kyoto Encyclopedia of Genes and Genomes (KEGG), and gene set enrichment analysis (GSEA). Somatic mutation data were downloaded from TCGA and analyzed using the maftools R package. Tumor Immune Estimation Resource (TIMER) and CIBERSORT were used to analyze correlations between *MTOR* and tumor-infiltrating immune cells (TIICs). Finally, we detected *MTOR* mutations in a CRC cohort from our database using whole-exome sequencing.

**Results:**

We found that *MTOR* was overexpressed in Asian CRC patients and associated with a poor prognosis. Enrichment analysis showed that *MTOR* was involved in metabolism, cell adhesion, and translation pathways in CRC. High *MTOR* expression was correlated with high tumor mutation burden (TMB) and several TIICs. Finally, we found that the mTOR signaling pathway was activated in CRC lines characterized by microsatellite instability (MSI), and the frequency of *MTOR* mutations was higher in MSI-high (MSI-H) patients than in microsatellite stable (MSS) patients.

**Conclusions:**

*MTOR* may represent a comprehensive indicator of prognosis and immunological status in CRC. The genomic signatures of *MTOR* may provide guidance for exploring the role of mTOR inhibitors in CRC.

**Supplementary Information:**

The online version contains supplementary material available at 10.1186/s12885-022-09901-w.

## Introduction

Mammalian target of rapamycin (mTOR), the protein product of the *MTOR* gene, is a serine/threonine kinase and an important downstream effector of the phosphoinositide 3-kinase (PI3K)/protein kinase B (AKT) pathway. In mammalian cells, the PI3K/AKT/mTOR pathway plays a crucial role in the regulation of cell survival, metabolism, growth, and protein synthesis in response to upstream signals during both normal physiological and abnormal pathological conditions [1]. This pathway represents one of the most deregulated signaling pathways in human cancer. mTOR is considered to serve as a master regulator of this signaling pathway, and recent findings have reported that mTOR activation plays a vital role in human cancer [2]. As reported, mTOR is aberrantly overactivated in more than 70% of cancers [3]. In lung cancer, the activation of the mTOR pathway is associated with a poor prognosis [4, 5]. In breast cancer, mTOR expression correlated with a worse prognosis, and p-mTOR was more commonly detected in triple-negative breast cancers [6, 7]. The abnormality of mTOR activation in cancer offers opportunities for targeted therapy. Several distinct classes of drugs have been developed to inhibit mTOR, including antibiotic allosteric mTOR inhibitors (rapamycin and its rapalogs), ATP-competitive mTOR inhibitors, and mTOR/PI3K dual inhibitors. Several mTOR inhibitors have reached various stages of clinical trials, but only temsirolimus and everolimus have been approved by the Food and Drug Administration for clinical use in the treatment of cancer patients. Preclinical studies have demonstrated the efficacy of most mTOR inhibitors in several types of cancers; however, early clinical trials have yielded mixed results because inhibitors (such as ATP-competitive mTOR inhibitors and mTOR/PI3K dual inhibitors) were often associated with dose-limiting toxicities, possibly due to low selectivity [8–12]. The degree of dependence on mTOR activation varies, and even among patients with similar mutation profiles, the outcomes associated with mTOR inhibitor therapy have been reported to vary across different cancer types. Although temsirolimus has been approved for the treatment of patients with advanced-stage renal cell carcinoma (RCC), clinical experience has indicated that only 8.6% of patients experienced an objective response in phase III Global advanced-stage RCC trial [13]. Identifying predictive biomarkers capable of determining which patients might benefit from mTOR inhibitors remains necessary. Mutations in *MTOR* have been shown to alter the sensitivity to rapalog treatment in several cancer types [14–16]. Therefore, the molecular alterations of *MTOR* warrant further investigation.

Colorectal cancer (CRC) is the second most common cause of cancer-related deaths worldwide [17] and represents one of the most common malignant tumors in China. The incidence and mortality of CRC in China ranked third and fifth, respectively, among all malignant tumors in 2015, associated with 388,000 new cases and 187,000 deaths [18]. Although developments in targeted therapy and immunotherapy have improved outcomes for some patients, the prognosis of CRC remains far from satisfactory for the majority of patients [19]. Therefore, the identification of potential prognostic biomarkers and novel treatment targets for CRC remains necessary. Several studies have examined the role of the PI3K/AKT/mTOR pathway in CRC, but most of these have focused on upstream regulators, such as phosphatase and tensin homolog (PTEN) and phosphatidylinositol-4,5-bisphosphate 3-kinase catalytic subunit alpha (PIK3CA), with few studies focused on mTOR itself. Previous studies [20–23] have demonstrated that mTOR is highly activated in CRC, and associated with the proliferation of CRC. However, the functional outcomes and the underlying activating mechanisms of MTOR in CRC remain to be investigated. Currently, the efficacy of mTOR inhibitors is limited in CRC, which may be related to the lack of study regarding *MTOR* function in this cancer type.

In this study, *MTOR* expression in CRC and the correlations between *MTOR* expression and prognosis were analyzed using datasets obtained from The Cancer Genome Atlas (TCGA) and Gene Expression Omnibus (GEO) databases. Differential gene expression analyses, including Gene Ontology (GO), Kyoto Encyclopedia of Genes and Genomes (KEGG) pathway enrichment analysis, and gene set enrichment analysis (GSEA), were used to investigate the possible molecular functions of *MTOR* in CRC. We also analyzed the correlation between *MTOR* and tumor mutational burden (TMB), the tumor immune microenvironment, and microsatellite instability (MSI) status. Finally, for further exploration and verification, we examined the single-nucleotide variations in the *MTOR* sequence among an Asian population cohort from our database. Our findings indicate the prognostic value of *MTOR* in CRC and demonstrate the potential associations between *MTOR* and tumor mutation. We hope this study will contribute to new prognostic monitoring and treatment strategies for CRC patients.

## Materials and methods

### Data acquisition and gene expressional analysis

The mRNA levels of *MTOR* in cancer and para-cancer tissues were analyzed through the “Differential Expression” module in Tumor Immune Estimation Resource (TIMER; https://cistrome.shinyapps.io/timer/) [24]. Gene expression data and corresponding clinical data for the CRC samples included in TCGA datasets (colon carcinoma [COAD] and rectal carcinoma [READ]) were obtained from the University of California Santa Cruz (UCSC, https://xenabrowser.net/datapages/). Corresponding somatic mutation data were obtained from the TCGA website (https://portal.gdc.cancer.gov/) [25]. In addition, we selected four datasets (GSE41657, GSE113513, GSE87211, and GSE75316) from the GEO database. The GSE41657 and GSE113513 datasets contain both CRC tissue and normal colon tissue samples from Asian patients. The GSE87211 dataset was used to perform a prognostic analysis of CRC patients. The GSE75316 dataset is comprised of microsatellite stable (MSS), MSI-high (MSI-H), and MSI-low (MSI-L) CRC patients.

### Overall survival analysis

Survival analyses in the TCGA and GSE87211 datasets were compared between high and low *MTOR* expression groups based on cutoff levels established at the median value (50%) and the quantile values (the top 25% and the bottom 25%) of *MTOR* expression. Kaplan–Meier curves were generated using the “survival” R package.

### Differentially expressed genes and functional enrichment analysis

Differentially expressed genes (DEGs) between the high and low *MTOR* expression groups (determined by quantile) in the TCGA dataset were identified using the limma R package with the parameters of |log_2_ fold change (log_2_FC)| > 0.5 and false discovery rate (FDR) < 0.05. For further functional enrichment analysis of DEGs, GO enrichment analysis, including cellular components (CC), molecular functions (MF), and biological processes (BP), and KEGG analysis were performed using the clusterProfiler R package and visualized by the OmicShare tools, a free online platform for data analysis (http://www.omicshare.com/tools). GSEA software (https://www.gsea-msigdb.org/gsea/login.jsp/) was also utilized to analyze the enriched pathways of identified DEGs.

### Mutation analysis in CRC

The maftools R package [26] was used to analyze and visualize the original MAF files of all CRC patients and some of the CRC patients from the high and low *MTOR* expression groups in TCGA. The maftools R package was also applied to visualize the mutation signatures of genes associated with the mTOR pathway. TMB was calculated as the number of somatic nonsynonymous variations in the TCGA datasets. The “Mafcompare” function in the maftools R package was utilized to identify differentially mutated genes between high and low *MTOR* expression groups.

### Characteristics of the tumor microenvironment

The “Gene” and “SCNA” modules of TIMER were applied to explore the correlations between *MTOR* and the abundance of six subtypes of immune cell infiltrates (B cells, CD4^+^ T cells, CD8^+^ T cells, neutrophils, macrophages, and dendritic cells) in CRC. Considering the important role of immune cells in the tumor microenvironment (TME), the CIBERSORT method (https://cibersort.stanford.edu/) [27] was used to further quantify the proportions of 22 immune infiltration cells in CRC samples between the high and low *MTOR* expression groups.

### CRC lines and cell culture

HCT-116, RKO, SW-620, and HT-29 cell lines were obtained from the American Type Culture Collection (ATCC, Manassas, VA). The microsatellite status of each cell line was identified using labeled primers for the co-amplification of five quasimonomorphic mononucleotide repeat markers (BAT-25, BAT-26, NR-21, NR-24, and MONO-27) obtained from AmoyDx Biotechnology Co., Ltd. (Xiamen, China). Consistent with existing literature reports [28], RKO and HCT-116 were classified as MSI-H, and HT-29 and SW-620 were classified as MSS. Cells were grown in Dulbecco’s modified Eagle’s medium/F12 medium supplemented with 10% fetal bovine serum and cultured in a humidified atmosphere containing 5% CO_2_ at 37 °C.

### Western blotting

Cells were lysed with a radioimmunoprecipitation assay buffer containing 1% phenylmethylsulfonyl fluoride. Insoluble materials were removed by centrifugation at 12,000 rpm for 15 min at 4 °C. The concentration of total protein was determined using the Pierce BCA Protein Assay Kit (Thermo Fisher Scientific, Waltham, USA). Proteins were separated by 10% sodium dodecyl sulfate-polyacrylamide gel electrophoresis, transferred onto polyvinylidene difluoride membranes, and probed with the appropriate antibodies, as indicated. An antibody against mTOR (Rabbit, catalog number sc-1549-R) was obtained from Santa Cruz Biotechnology (Santa Cruz, CA, USA), and an antibody against p-mTOR (Rabbit, catalog number SAB4504476) was obtained from Sigma-Aldrich. An anti-glyceraldehyde 3-phosphate dehydrogenase (GAPDH; Mouse, catalog number 60004-1-Ig) was purchased from Proteintech (Chicago, IL, USA). The secondary antibodies included anti-mouse (catalog number A4416) and anti-rabbit (catalog number A6154) antibodies obtained from Sigma-Aldrich.

### Cytotoxicity experiments

For cytotoxicity experiments, cell lines were seeded in a 96-well plate and treated with rapamycin (Med Chem Express, USA) for 72 h. Rapamycin was dissolved in dimethylsulfoxide to a final concentration of 10 mM. The working concentrations were diluted to 0, 10, 15, 20, 25, and 30 μM, and four wells were used for each concentration. Cell proliferation was assessed using the Cell Counting Kit-8 (CCK-8; Meilunbio Biotechnology Co., Ltd). The absorbance was measured at 450 nm using a model 3550 microplate reader (BioRad Laboratories, Inc., Hercules, CA, USA). Half maximal inhibitory concentration (IC_50_) values were calculated, and the inhibition curve was plotted using GraphPad Prism software, Version 8.0.

### Patients and tissue samples

Following the guidelines set by the Ethical Committee of Ruijin Hospital, 74 CRC cases were recruited from Ruijin Hospital (Shanghai, China). Clinicopathological data were retrospectively collected, comprising sex, age, tumor location, pathological tumor node metastasis stage, vascular invasion, and MLH1, PMS2, MSH2, and MSH6 expression (positive or negative). Informed consent was obtained from all patients before the study. The CRC stages were categorized according to Union for International Cancer Control guideline (8th Edition).

For the tissue microarray, the cohort of 74 tumor tissues and paired normal colonic tissues were fixed with formaldehyde and embedded with paraffin. A tissue microarray was constructed for further immunohistological assays. Microscopy images were observed using the Biological Microscope (Elipse Ci-L, Nikon, Japan) and captured using Digital Pathology Slide Scanner (KF-PRO-120, Konfoong Biotech International Co., Ltd., China). The measured resolution of microscopy images was 0.25 μm/pixel. Based on the German semi-quantitative scoring system, the staining score for each tissue was evaluated by two independent pathologists, and a score greater than 3 was considered a positive expression.

### DNA and whole-exome sequence

For whole-exome sequencing, DNA was isolated from 48 frozen tumor tissues collected from a previous cohort. DNA was isolated using standard extraction methods (Qiagen, Valencia, CA) and quantified using PicoGreen-based dsDNA detection (Life Technologies, Carlsbad, CA). Indexed sequencing libraries were prepared from 500 ng sonically sheared DNA samples using Illumina TruSeq LT reagents (Illumina Inc., San Diego, CA).

Using a custom DNA bait set created by IDT (Integrated DNA Technologies, Coralville, Iowa), libraries were enriched using solution-based hybrid capture. The DNA bait set included a target panel that covered the whole region of the *MTOR* gene, which encompasses 160,017 bp of the genome. Massively parallel sequencing was performed using an Illumina Novaseq6000 (Illumina) with paired-end 150 bp (PE150) reads.

Pooled sample reads were deconvoluted (demultiplexed) and sorted using Picard version 2.24.2 and later versions (Broad Institute, Cambridge, MA). Reads were aligned to the reference sequence hg19, obtained from the Human Genome Reference Consortium, using BWA version 0.7.17. Duplicate reads were identified and removed using Picard. The median mean target coverage per sample after the removal of duplicate reads was 1224×. The alignments were further refined using the Genome Analysis Toolkit version 4.1.1.0 and later versions (Broad Institute) for localized realignment around insertion and deletion (indel) sites. The recalibration of quality scores was performed using the Genome Analysis Toolkit. Mutation analysis for single-nucleotide variants was performed using MuTect (Broad Institute). Indels were called using Indelocator (Broad Institute). Integrative Genomics Viewer version 2.0.16 or later versions (Broad Institute) was used to visualize and interpret data. Variants were filtered to exclude synonymous variants, known germline variants in the Single-Nucleotide Polymorphism database, and variants that occur at a population frequency of > 0.1% in the Exome Sequencing Project database. Copy number detection was performed by analysis of fractional coverage of a defined genomic interval compared with pooled normal samples. The structural variant analysis was performed using Delly to detect larger indels. Finally, 10 cases were excluded from analysis due to disqualification of sequencing data, and 38 patients were selected for the final analysis.

### Statistical analysis

R software was utilized for statistical analysis. The Pearson Chi-square test was used to analyze the association between *MTOR* mutations and clinical characteristic variables. A *p*-value < 0.05 was considered significant.

## Results

### *MTOR* expression analysis in the pan-cancer cohort indicated overexpression in CRC

**Fig. 1:**
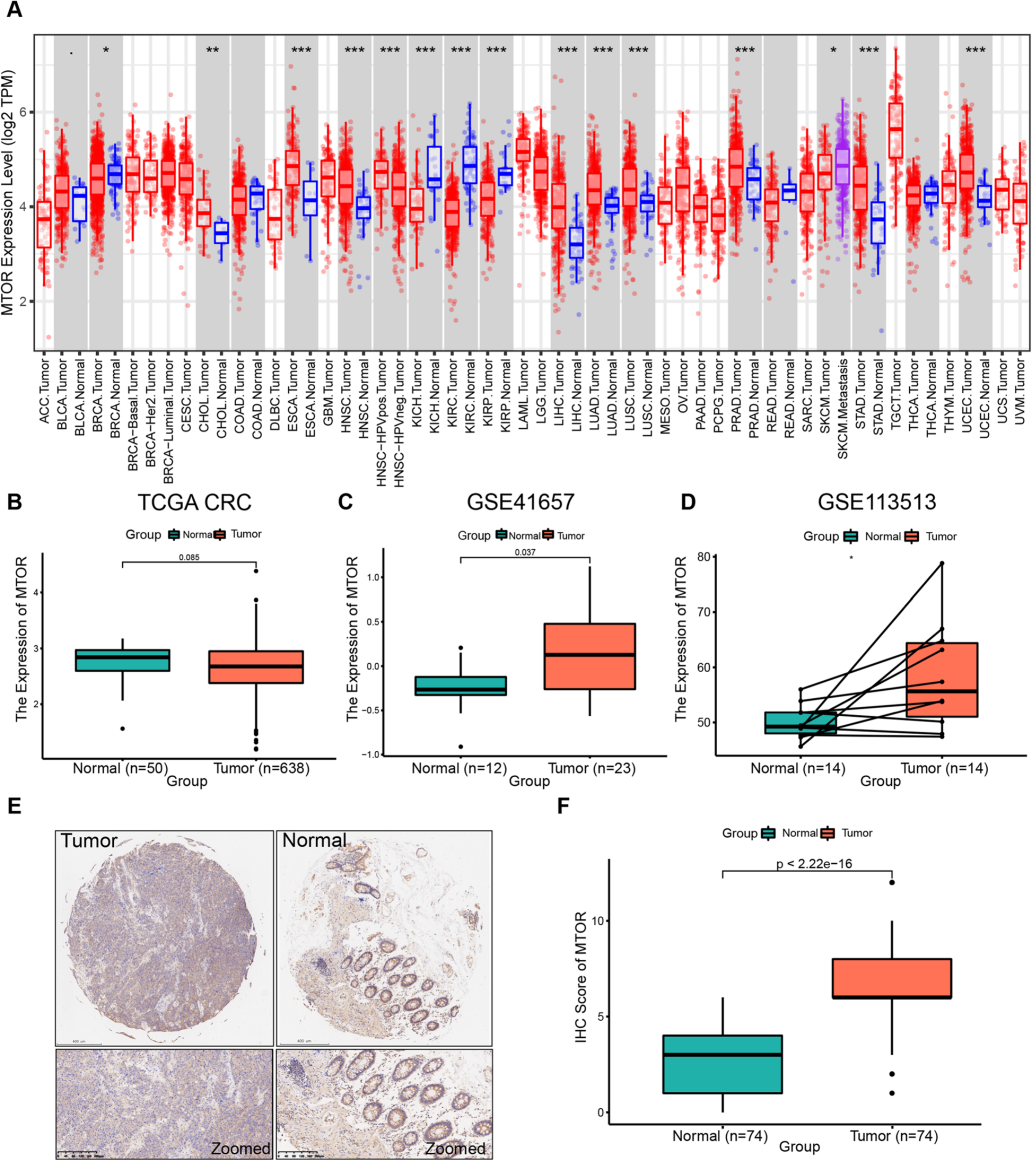
Expression analysis of *MTOR* in cancers. **A** The *MTOR* expression levels in 15 out of 33 categories of human cancers differed from normal tissues. *p < 0.05, **p < 0.01, ***p < 0.001. The blue boxes represent normal tissues; the red boxes represent tumor tissues; the purple boxes represent metastatic tumor tissues. ACC, Adrenocortical carcinoma; BLCA, Bladder urothelial carcinoma; BRCA, Breast invasive carcinoma; CESC, Cervical squamous cell carcinoma and endocervical adenocarcinoma; CHOL, Cholangiocarcinoma; COAD, Colon adenocarcinoma; DLBC, Lymphoid neoplasm diffuse large b-cell lymphoma; ESCA, Esophageal carcinoma; GBM, Glioblastoma multiforme; HNSC, Head and neck squamous cell carcinoma; KICH, Kidney chromophobe; KIRC, Kidney renal clear cell carcinoma; KIRP, Kidney renal papillary cell carcinoma; LAML, Acute myeloid leukemia; LGG, Brain lower grade glioma; LIHC, Liver hepatocellular carcinoma; LUAD, Lung adenocarcinoma; LUSC, Lung squamous cell carcinoma; MESO, Mesothelioma; OV, Ovarian serous cystadenocarcinoma; PAAD, Pancreatic adenocarcinoma; PCPG, Pheochromocytoma and paraganglioma; PRAD, Prostate adenocarcinoma; READ, Rectum adenocarcinoma; SARC, Sarcoma; SKCM, Skin cutaneous melanoma; STAD, Stomach adenocarcinoma; TGCT, Testicular germ cell tumors; THCA, Thyroid carcinoma; THYM, Thymoma; UCEC, Uterine corpus endometrial carcinoma. UCS, Uterine carcinosarcoma; UVM, Uveal melanoma. **B** *MTOR* expression levels in CRC and corresponding normal tissues from TCGA. **C** Different *MTOR* expression levels between CRC and unpaired normal tissues from GSE41657. **D** Different *MTOR* expression levels between CRC and paired normal tissues from GSE113513. *p < 0.05. The GSE41657 and GSE113513 datasets are from Asian patients. **E** Representative immunohistochemistry (IHC) staining shows differences in mTOR expression between CRC and paired normal tissues from the Ruijin cohort. **F** The box plot displays the IHC scores for mTOR in CRC and paired normal tissues from the Ruijin cohort. CRC, colorectal cancer; TCGA, The Cancer Genome Atlas; mTOR, mammalian target of rapamycin

We first conducted a pan-cancer analysis of *MTOR* expression (Fig. 1A), and the TIMER analysis results indicated that compared with normal tissues, *MTOR* was downregulated in breast carcinoma (BRCA), clear cell RCC (KIRC), clear cell papillary RCC (KIRP), and chromophobe RCC (KICH), whereas *MTOR* was upregulated in cholangiocarcinoma (CHOL), esophageal carcinoma (ESCA), head and neck squamous cell carcinoma (HNSC), hepatocellular carcinoma (HCC), lung adenocarcinoma (LUAD), lung squamous cell carcinoma (LUSC), pancreatic adenocarcinoma (PAAD), gastric adenocarcinoma (STAD), and uterine corpus endometrial carcinoma (UCEC). The expression levels of *MTOR* in bladder urothelial carcinoma (BLCA), colon carcinoma (COAD), rectal carcinoma (READ), and thyroid carcinoma (THCA) did not differ from that in normal tissues. These results demonstrated that *MTOR* was abnormally expressed in multiple tumor types.

We determined the expression level of *MTOR* in CRC samples obtained from multiple TCGA datasets (TCGA-COAD combined with TCGA-READ). However, no difference in *MTOR* expression levels was observed between CRC and normal tissues (Fig. 1B). Ethnic differences can influence tumorigenesis; therefore, we selected CRC expression data obtained from an Asian population in the GEO database for further analysis. The expression of *MTOR* was significantly increased in unpaired CRC samples compared with normal tissues in the GSE41657 cohort (*p* = 0.037; Fig. 1C). The trend toward upregulation was also observed between paired CRC samples and normal samples in the GSE113513 cohort (*p* < 0.05; Fig. 1D). Moreover, we collected 74 CRC tumor tissues and paired normal mucosal tissue samples from patients with CRC for immunohistochemistry validation. The results showed that *MTOR* was significantly overexpressed in CRC tissues compared with normal mucosal tissues (*p* < 0.05; Fig. 1E and F).

**Fig. 2:**
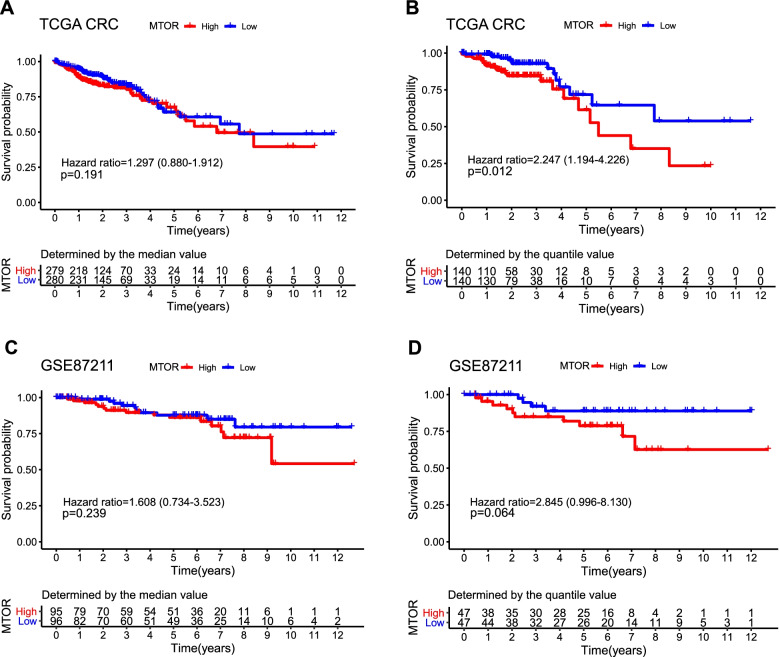
Prognostic value of *MTOR* in CRC patients. **A**, **B** Kaplan–Meier survival curve comparing high (*N* = 279) and low (*N* = 280) *MTOR* expression determined by the median value and high (*N* = 140) and low (*N* = 140) *MTOR* expression determined by quantile value for the TCGA CRC patient cohort. **C**, **D** Kaplan–Meier survival curve comparing high (*N* = 95) and low (*N* = 96) *MTOR* expression determined by the median value and high (*N* = 47) and low (*N* = 47) *MTOR* expression determined by quantile value for the CRC patients in the GSE87211 database. CRC, colorectal cancer; TCGA, The Cancer Genome Atlas

Based on *MTOR* overexpression in Asian CRC populations, we further analyzed its prognostic value. First, we established low and high *MTOR* expression groups according to the median *MTOR* expression value. As indicated in Fig. 2A and C, no significant difference in prognosis between the two groups was observed in TCGA and GSE87211 datasets. We set the cutoff levels according to the quantile value (top 25% and bottom 25%) to establish low and high *MTOR* expression groups. As shown in Fig. 2B, higher *MTOR* expression was significantly related to worse overall survival in the TCGA dataset (*p* = 0.012, hazard ratio = 2.247, 95% confidence interval = 1.194–4.226). In the GEO database (Fig. 2D), although no significant difference was observed between the two groups, a trend toward a worse prognosis was observed for the high *MTOR* expression group (*p* = 0.064, hazard ratio = 2.845, 95% confidence interval = 0.996–8.130).

### Predicted *MTOR* functions and pathways in CRC

**Fig. 3:**
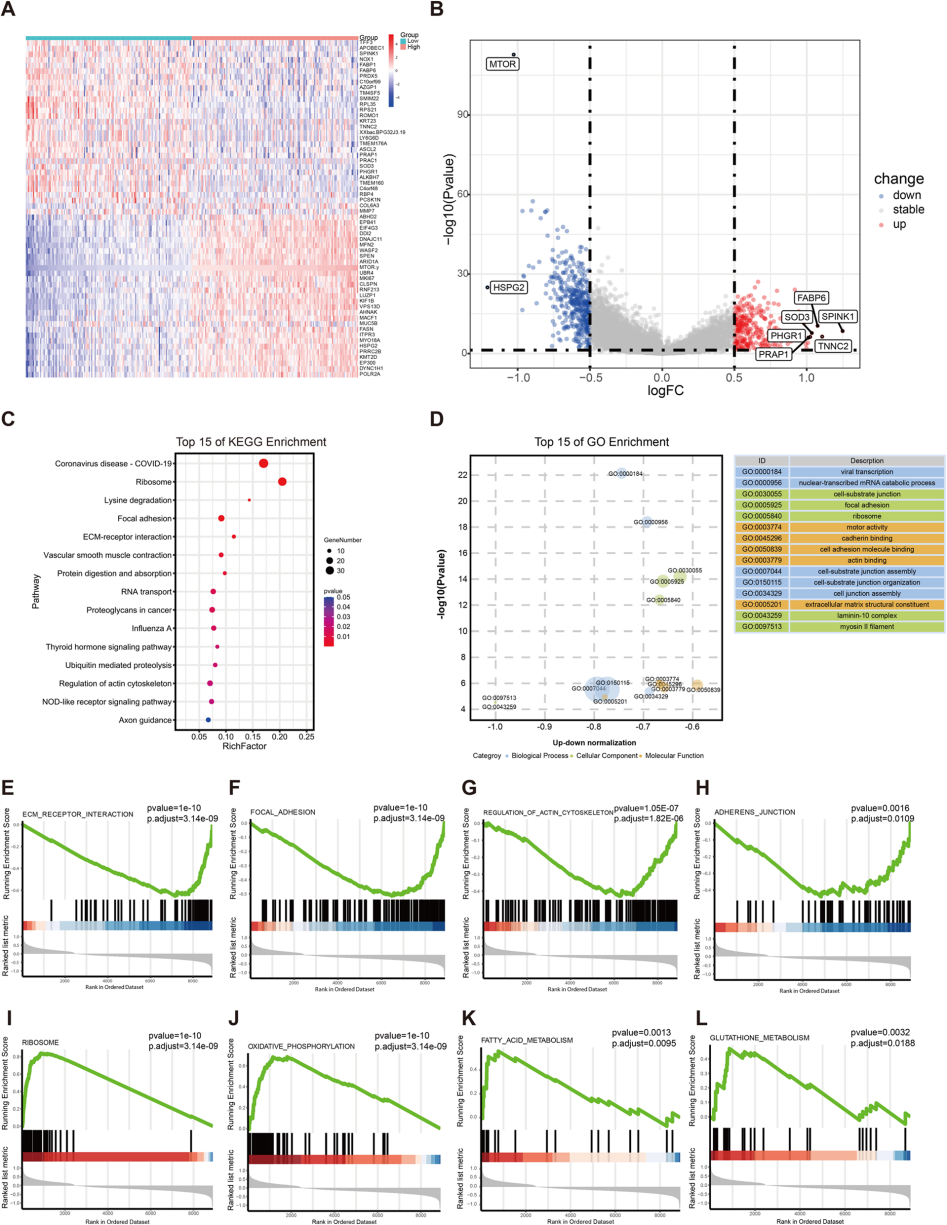
Identification of *MTOR*-related DEGs in CRC and enrichment pathway analysis. **A**, **B** The heatmap and volcano plot show DEGs between low and high *MTOR* expression groups in CRC patients; “down” and blue dots indicate downregulated DEGs; “up” and red dots indicate upregulated DEGs; “stable” indicates that the difference was not significant. **C** The top 15 pathways involved in DEGs by KEGG. **D** The top 15 functions involved in DEGs by GO enrichment analysis. The GSEA results show differentially enriched terms involved in DEGs. **E** “ECM-receptor interaction”, **F** “focal adhesion”, **G** “regulation of actin cytoskeleton”, **H** “adherens junction”, **I** “ribosome”, **J** “oxidative phosphorylation”, **K** “fatty acid metabolism”, and (**L**) “glutathione metabolism”. DEGs, differentially expressed genes; CRC, colorectal cancer; KEGG, Kyoto Encyclopedia of Genes and Genomes; GO, Gene Ontology; GSEA, gene set enrichment analysis

To clarify the underlying mechanism of *MTOR* in the promotion of CRC formation, we performed DEG analysis between high (*N* = 123) and low *MTOR* expression patients (*N* = 117, determined by quartile) from the TCGA dataset. The heatmap and the volcano plot in Fig. 3A and B display the up- and downregulated DEGs.

We also performed GO and KEGG pathway enrichment analyses on the DEGs. KEGG enrichment analysis showed that the DEGs were closely linked to a variety of pathways involved in viral transcription, cell adhesion, and translation (Fig. 3C). The GO analysis revealed several MF terms, including motor activity, cell adhesion molecule binding, cadherin binding, actin binding, and extracellular matrix structural constituents. GO BP terms indicated that DEGs were involved in viral transcription, nuclear-transcribed mRNA catabolic processes, cell-substrate junction assemblies, cell-substrate junction organizations, and cell junction assemblies. GO CC terms indicated various cellular structures, including cell-substrate junction, focal adhesion, ribosome, lamini-10 complex, and myosin II filament (Fig. 3D). Both GO and KEGG analyses identified *MTOR* as significantly correlated with cell adhesion and metabolism; therefore, we further explored the associations between *MTOR* and cell adhesion and translation through GSEA (Fig. 3E–L). Based on the validation dataset, we identified DEGs involved in metabolism pathways, including oxidative phosphorylation, fatty acid metabolism, and glutathione metabolism. These results showed that *MTOR* might affect these signaling pathways, contributing to a poor prognosis in CRC patients.

### Relationship between *MTOR* mRNA expression and somatic variants in CRC

**Fig. 4:**
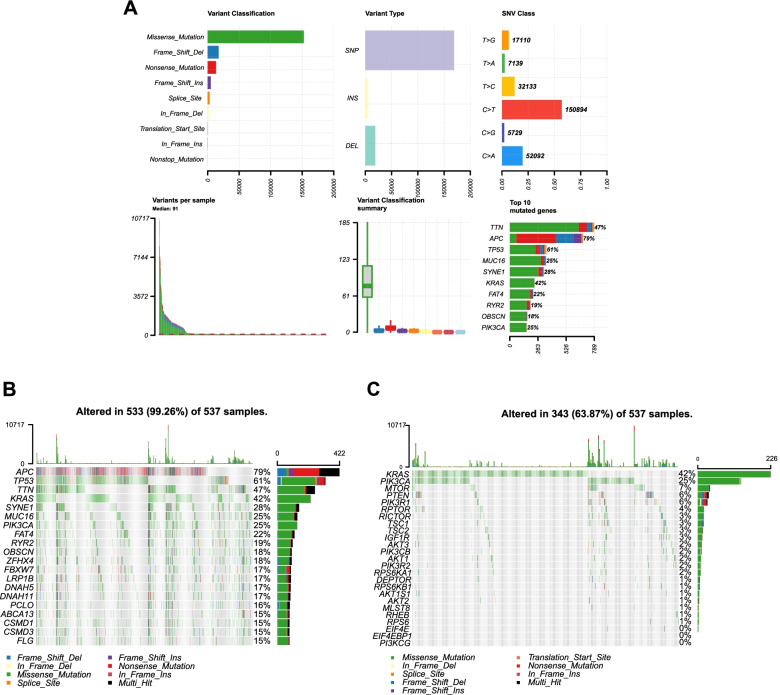
Somatic mutation profiles in CRC. **A** Cohort summary plot of the mutation information among 537 CRC patients from TCGA displaying the distribution of variants according to variant classification, type, and SNV class. The bottom part (from left to right) indicates the mutation load for each sample, and variant classification type. A stacked barplot shows the top ten mutated genes. **B** The oncoplot displays the mutation information of the CRC cohort. Genes are ordered by their mutation frequency. **C** The oncoplot shows the mutation information of genes associated with the mTOR pathway. CRC, colorectal cancer; TCGA, The Cancer Genome Atlas, mTOR, mammalian target of rapamycin

CRC is characterized by high levels of genetic mutation, which plays an important role in the response to therapy. To identify the somatic mutations among the 537 CRC patients in the TCGA database, mutation data were downloaded, and a summary of the mutation information was visualized in Fig. 4A and B. The top 10 mutated genes in CRC by percentage are visualized, including *APC* (79%), *TP53* (61%), *TTN* (47%), *MUC16* (25%), *SYNE1* (28%), *KRAS* (42%), *FAT* (22%), *RYR2* (19%), *OBSCN* (18%), and *PI3KCA* (25%). Oncoplots displayed significantly mutated genes in the CRC cohort, which were sorted by mutational frequency. In addition, we explored the mutation information associated with mTOR pathway-related genes in CRC and found that the mutation frequency of *MTOR* was only inferior to the mutation frequencies of *KRAS* and *PI3KCA* (Fig. 4C).

**Fig. 5:**
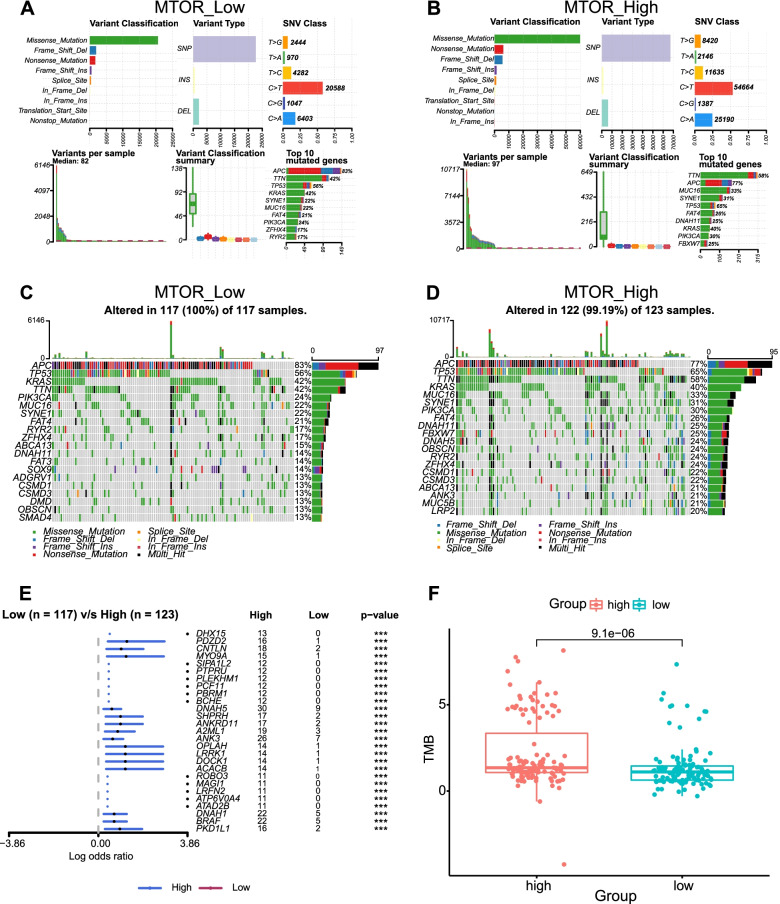
Statistics describing mutation and TMB between low and high *MTOR* expression groups. **A**, **B** Cohort summary plots of the low and high *MTOR* expression groups displaying the distribution of variants according to variant classification, type and SNV class. The bottom part (from left to right) indicates the mutation load for each sample, and variant classification type. A stacked barplot shows the top ten mutated genes. **C**, **D** The oncoplots show the different mutation information between the low and high *MTOR* expression groups. **E** The forest plot displays differentially mutated genes between the high and low *MTOR* expression groups. Bars indicate the 95% confidence interval for the odds ratio. The adjacent table includes the number of patients in the high and low *MTOR* expression groups with the mutations in the highlighted gene. ***p < 0.001. **F** The boxplot shows differences in TMB between high and low *MTOR* expression groups. TMB, tumor mutational burden; CRC colorectal cancer

Next, we assessed the distribution of somatic variants in CRC between the low and high *MTOR* expression groups (determined by quartile). No differences in classification were observed between the two groups, but the number of altered bases in each sample was significantly higher for the high *MTOR* expression group than for the low *MTOR* expression group (Fig. 5A and B). Analysis of the mutation annotation files for the TCGA cohort revealed that the mutation frequencies for the top 20 driver genes were significantly different between the low and high *MTOR* expression groups (Fig. 5C and D). In the high expression group, the mutation frequencies for the top 20 genes increased substantially, with all being greater than 20%, whereas, in the low expression group, only eight genes had mutation frequencies greater than 20%. We also positioned the TMB scores of the two groups within a pan-cancer TMB score scatter diagram, which revealed that the TMB of the high *MTOR* expression group was higher than the average TMB level of rectal cancer, whereas the TMB of the low *MTOR* expression group was lower than that of rectal cancer (Supplementary Fig. 1A and B). We also analyzed components of the affected pathways, including RAS, WNT, NOTCH, Hippo, MYC, TGF-beta, and other pathways associated with the development of colorectal cancer, and compared them between the low and high *MTOR* expression groups (Supplementary Fig. 1C and D). The mutational frequencies among the components of these pathways increased in the high *MTOR* expression group. We also employed the mafCompare function to identify differentially mutated genes between low and high *MTOR* expression groups. Comparison between the low and high *MTOR* expression groups revealed 27 genes that were differentially mutated (*p* < 0.001; Fig. 5E). All of the identified genes were significantly enriched in the high *MTOR* expression group. The comparison of TMB between the two groups showed that the TMB level in the *MTOR* high expression group was higher than that of the low *MTOR* expression group (*p* < 0.001; Fig. 5F). These results indicated that *MTOR* was closely related to tumor mutations.

### Relationship between *MTOR* and the tumor-infiltrating immune cells (TIICs) in CRC

**Fig. 6:**
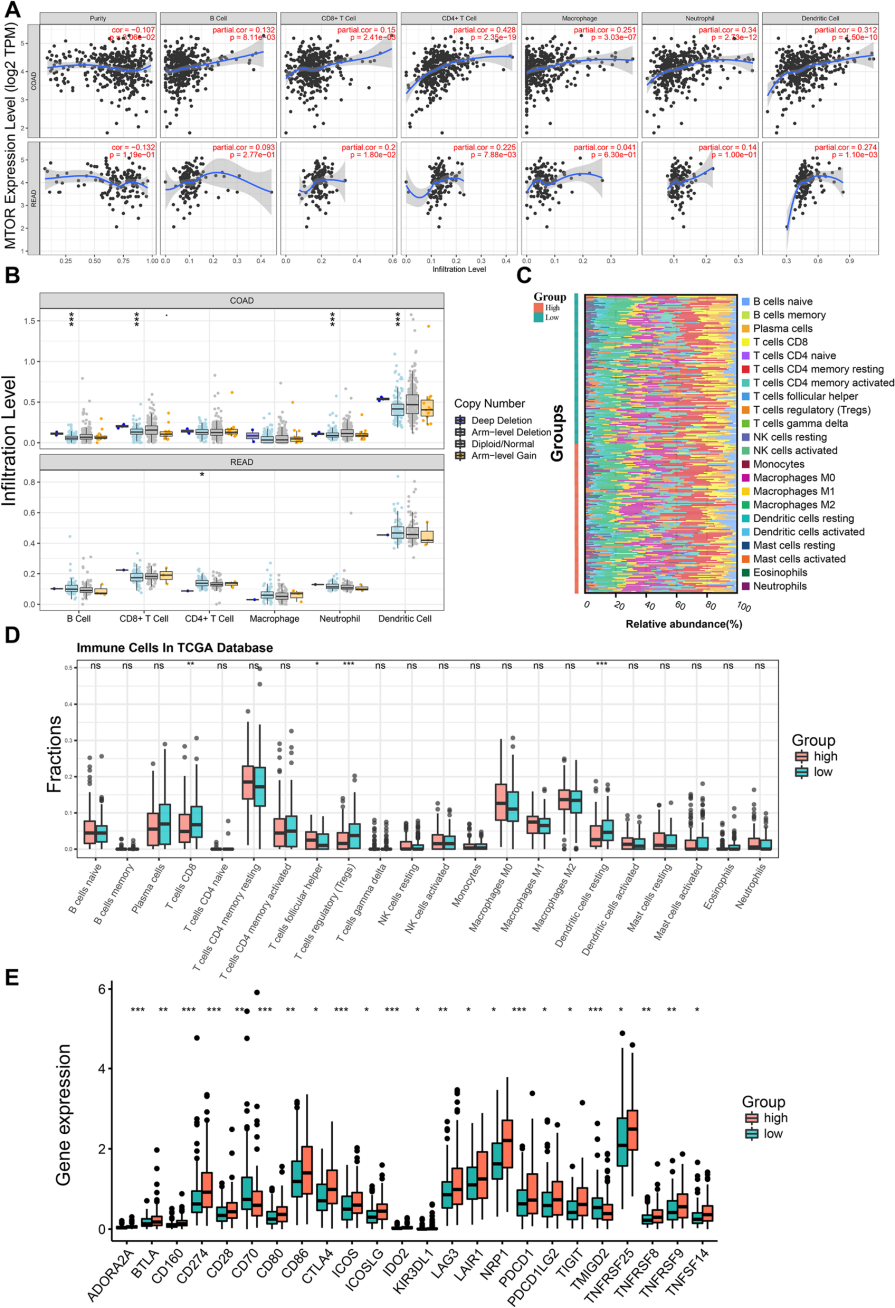
Correlation analysis between *MTOR* expression and TIICs in CRC. **A** The scatterplots display the correlations between *MTOR* expression and infiltrating B cells, CD8^+^ T cells, CD4^+^ T cells, macrophages, neutrophils and dendritic cells in COAD and READ. **B** The boxplot shows the correlation between *MTOR* copy number variation and infiltrating B cells, CD8^+^ T cells, CD4^+^ T cells, macrophages, neutrophils and dendritic cells in COAD and READ. *p < 0.05, ***p < 0.001. **C** The stacked bar chart shows the different proportion of TIICs between high and low *MTOR* expression groups in CRC. **D** The box plot shows the different fraction of TIICs between high and low *MTOR* expression groups in CRC. ns, not significant, *p < 0.05, **p < 0.01, ***p < 0.001. **E** The box plot shows differences in immune checkpoint genes expression between high and low *MTOR* expression groups in CRC. *p < 0.05, **p < 0.01, ***p < 0.001. CRC, colorectal cancer; TIICs, tumor-infiltrating immune cells; COAD, Colon adenocarcinoma; READ, Rectum adenocarcinoma

We employed TIMER to explore the relationships between *MTOR* expression and the infiltration levels of six immune cell subtypes in the CRC immune microenvironment. As shown in Fig. 6A, *MTOR* expression was significantly correlated with the infiltration levels of B cells (*p* = 8.11e− 03; *p* = 2.77e− 01), CD4^+^ T cells (*p* = 2.35e− 19; *p* = 7.88e− 03), CD8^+^ T cells (*p* = 2.41e− 03; *p* = 1.80e− 02), neutrophils (*p* = 2.73e− 12; *p* = 1.00e− 01), macrophages (*p* = 3.03e− 07; *p* = 6.30e− 01), and dendritic cells (*p* = 1.50e− 10; *p* = 1.10e− 03) in the COAD and READ databases, respectively. These results confirmed that *MTOR* expression was associated with the CRC immune microenvironment. To elucidate the potential mechanisms between *MTOR* expression and different immune cell infiltrations, we further analyzed the effects of somatic cell *MTOR* copy number alterations (CNAs) on immune cell infiltration (Fig. 6B). The *MTOR* CNAs included deep deletion, arm-level deletion, diploid/normal, and arm-level gain. Compared with diploid/normal, a decrease in the *MTOR* gene copy number (arm-level deletion) significantly alleviated the infiltration levels of B cells (*p* < 0.001), CD8^+^ T cells (*p* < 0.001), neutrophils (*p* < 0.001), and dendritic cells (*p* < 0.001) in COAD. In READ, arm-level deletion in the *MTOR* gene copy number was associated with an increased infiltration level of CD4^+^ T cells (*p* < 0.05). These results demonstrated that *MTOR* had pivotal regulatory effects on the TME in CRC patients.

We used CIBERSORT to estimate the immune infiltration landscape of the TME by exploring the biological role of *MTOR* in the TME. The proportions of tumor-infiltrating immune cells (TIICs) in the TME varied significantly between low and high *MTOR* expression groups (Fig. 6C). The results further showed that CD8^+^ T cells, T regulatory (Treg) cells, follicular helper T cells, and resting dendritic cells were the primary immune cells affected by *MTOR* expression (Fig. 6D). Among these, CD8^+^ T cells (*p* < 0.01), resting dendritic cells (*p* < 0.001), and Treg cells (*p* < 0.001) exhibited lower proportions in the high *MTOR* expression group than in the low *MTOR* expression group. Conversely, the proportion of follicular helper T cells (*p* < 0.05) was significantly upregulated in the high *MTOR* expression group. In addition, we investigated the differences in the expression levels of immune checkpoints between the two groups (Fig. 6E). The results showed that expression of *PDCD-1* (*PD-1*), *CTLA4*, *LAG3*, *TNFRSF8/9/14/25*, *IDO2* et al. were significantly different.

### Relationship between *MTOR* and MSI

**Fig. 7:**
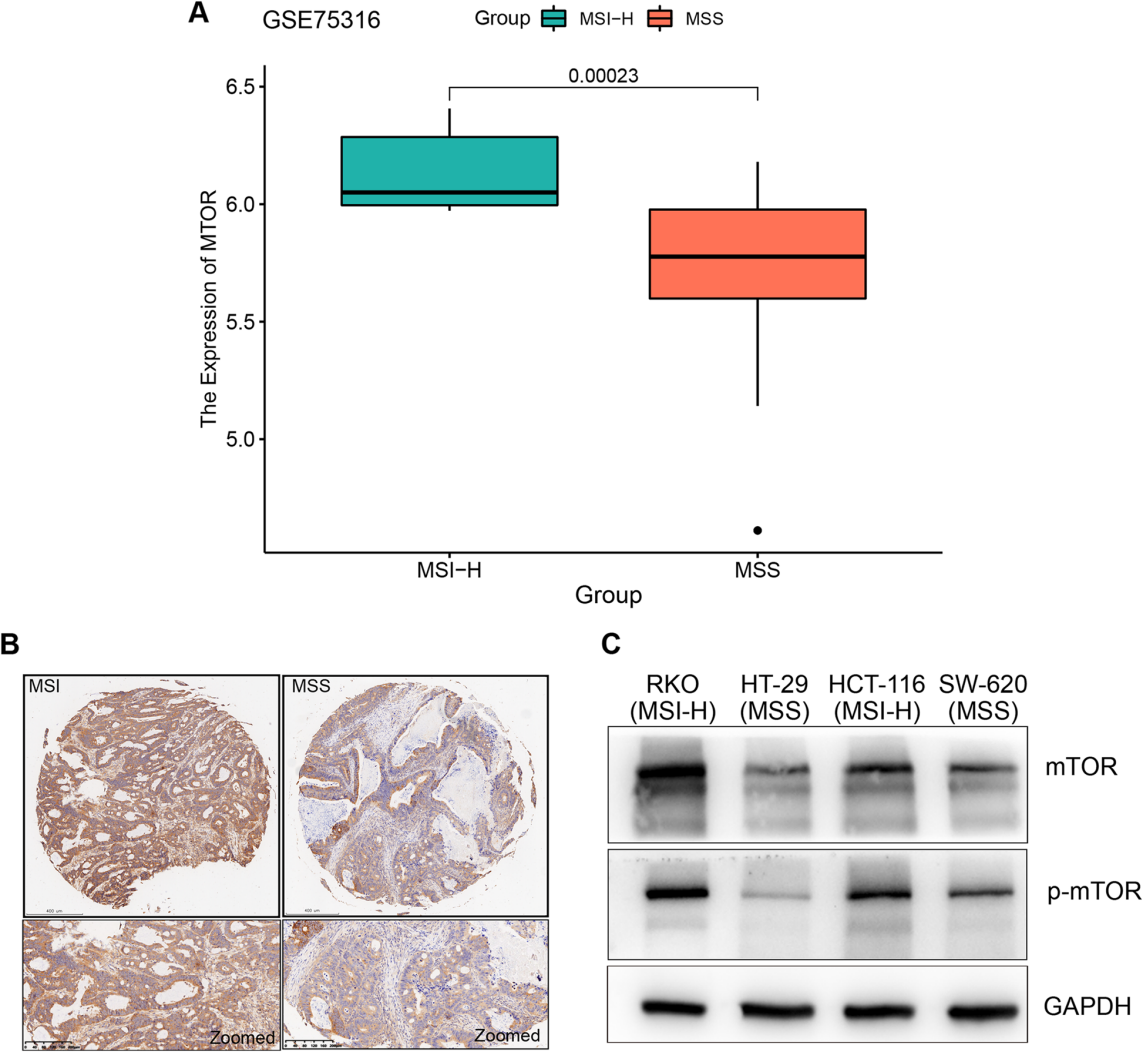
Expression analysis of *MTOR* in MSI and MSS CRC. **A** The boxplot shows the different *MTOR* expression levels between MSI-H and MSS CRC patients from GSE75316. **B** Representative IHC staining shows differences in mTOR expression between MSI and MSS CRC tissues. **C** The western blots show protein expression levels of mTOR and p-mTOR in CRC cell lines. The MSI cell lines RKO and HCT-116 and the MSS cell lines HT-29 and SW-620 are shown. Full-length blots are presented in Supplementary Figs. 3, 4, 5. CRC, colorectal cancer; MSI, microsatellite instability; MSI-H, MSI-high; MSS; microsatellite stable; mTOR, mammalian target of rapamycin

In addition to the comparison between CRC and normal tissue, we found that the expression of *MTOR* in the MSI subgroup was higher than that in the MSS subgroup (Fig. 7A). Analysis of mTOR protein levels using immunohistochemistry in CRC tissues revealed higher expression levels in the MSI samples than in the MSS samples (Fig. 7B). We performed western blot analysis to observe mTOR protein levels in different MSI and MSS CRC cell lines. The results showed that the protein levels of mTOR and p-mTOR were higher in MSI cell lines than in MSS cell lines (Fig. 7C). To investigate whether the activity of mTOR was changed in MSI CRC cell lines, we treated both MSI and MSS cell lines with rapamycin. As shown in Supplementary Fig. 2, cytotoxicity experiments revealed that rapamycin preferentially targeted MSI cell lines. MSS cell lines showed 2.4-fold and 1.4-fold higher IC_50_ values for rapamycin compared with the rapamycin IC_50_ values observed in MSI cell lines (22.68 vs. 9.57 μM, 22.68 vs. 16.01 μM, respectively). These results indicated that the activity of the mTOR signaling pathway might be altered in MSI CRC.

### Mutation signatures of *MTOR* in CRC

**Fig. 8:**
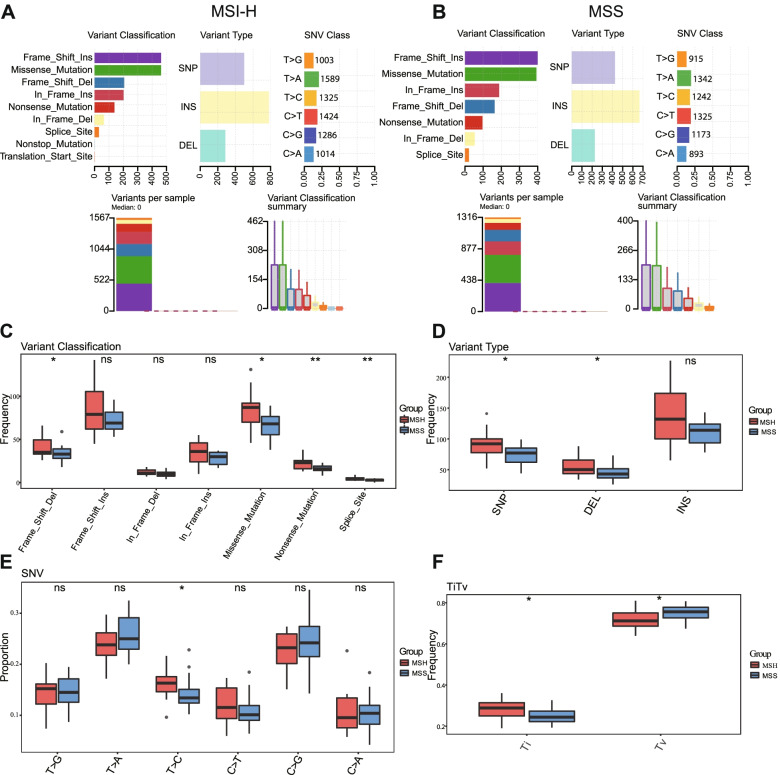
Mutation profiles of *MTOR* in MSI and MSS CRC. **A**, **B** Cohort summary plots of the MSI-H and MSS CRC patients displaying the distribution of variants in *MTOR* according to variant classification, type and SNV class. The bottom part (from left to right) indicates the mutation load for each sample, and variant classification type. **C** The differences in the distribution of variant classification among *MTOR* mutations between MSI and MSS CRC patients. **D** The differences in the distribution of variant type among *MTOR* mutations between MSI and MSS CRC patients. **E** The differences in the distribution of SNVs among *MTOR* mutations between MSI and MSS CRC patients. **F** The differences in the distribution of transition (Ti) and transversion (Tv) among *MTOR* mutations between MSI and MSS CRC patients. ns, not significant, *p < 0.05, **p < 0.01. CRC, colorectal cancer; MSI, microsatellite instability; MSI-H, MSI-high; MSS; microsatellite stable; SNV, single-nucleotide variant

Our results revealed that *MTOR* was a highly mutated gene in CRC (Fig. 4C). *MTOR* mutations have been shown to abnormally activate the mTOR pathway and increase sensitivity to rapamycin [29]. To analyze *MTOR* mutations in CRC, we collected frozen tumor tissues from 48 patients with CRC and conducted whole-exome sequencing of *MTOR* genes. The clinicopathological data of this patient cohort are shown in Table 1. Ten cases were excluded from analysis due to disqualification of sequencing data. A total of 38 patients were able to be analyzed, including 15 MSI-H patients and 23 MSS patients. *MTOR* mutations were identified by sequencing, classified, and analyzed. Among the classification categories, frameshift insertions and missense mutations accounted for the largest percentages of mutations, insertions occurred more frequently than single-nucleotide polymorphisms (SNP) or deletions, and T > A was the most common type of SNP in both the MSI-H and MSS groups (Fig. 8A and B). The frequencies of different mutational classes in *MTOR* were higher in the MSI-H group than in the MSS group (Fig. 8C–F). Among the variant classifications, the frequencies of frameshift deletions (*p* < 0.05), missense mutations (*p* < 0.05), nonsense mutations (*p* < 0.01), and splice sites (*p* < 0.01) were significantly increased in the MSI-H group compared with those in the MSS group. Among the variant types, the frequencies of SNPs (*p* < 0.05) and deletions (*p* < 0.05) were positive correlated with MSI. The analysis of SNPs showed that the proportion of T > C transitions (*p* < 0.05) was significantly higher in the MSI-H group than in the MSS group. In the MSI-H group, the frequencies of all transitions were significantly higher than in the MSS group (*p* < 0.05), whereas the frequencies of transversions were lower than in the MSS group (*p* < 0.05). These results indicated that the mutation frequency of *MTOR* was higher among MSI-H patients than among MSS patients.

**Table 1:** Baseline characteristic of patients with CRC from Ruijin Hospital (*N* = 48)

Factors	Cohort (***N*** = 48)
**Gender**	
Male	29 (60%)
Female	19 (40%)
**Age**	
< 60 y	15 (31%)
> 60 y	33 (69%)
**MS status**	
MSI-H	17 (35%)
MSS	31 (65%)
**Tumor location**	
Right	17 (35%)
Left	31 (65%)
**T stage**	
T2	3 (6%)
T3	23 (48%)
T4	22 (46%)
**N stage**	
N0	36 (75%)
N1-2	12 (25%)
**M stage**	
M0	40 (83%)
M1	8 (17%)
**Stage**	
I	3 (6%)
II	30 (63%)
III	8 (17%)
IV	7 (14%)
**Vascular invasion**	
positive	10 (21%)
negative	38 (79%)

**Fig. 9:**
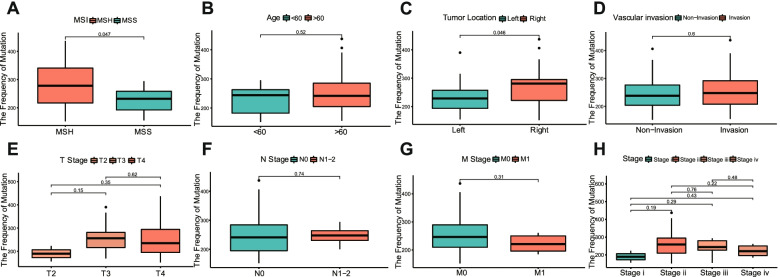
Correlation analysis between *MTOR* mutation and clinical characteristics. **A** Correlation of *MTOR* mutation frequency with MSI status. **B** Correlation of *MTOR* mutation frequency with age. **C** Correlation of *MTOR* mutation frequency with tumor location. **D** Correlation of *MTOR* mutation frequency with vascular invasion. **E** Correlation of *MTOR* mutation frequency with T stage. **F** Correlation of *MTOR* mutation frequency with N stage. **G** Correlation of *MTOR* mutation frequency with M stage. **H** Correlation of *MTOR* mutation frequency with clinicopathological stages. MSI, microsatellite instability; T, tumor; M, metastasis; N, node

We also analyzed the relationship between *MTOR* mutations and clinicopathological signatures. In addition to a significant increase in the MSI-H group (*p* = 0.047, Fig. 9A), the mutation frequency of *MTOR* differed according to tumor location. The mutation frequency of *MTOR* was higher in patients with right-sided CRC than in those with left-sided CRC (*p* = 0.046, Fig. 9C). A trend toward differences between *MTOR* mutation frequencies according to TN classification and tumor stage was observed, although no significant differences were identified, which may be due to the small sample size (Fig. 9E, F, and H). No significant differences in *MTOR* mutation frequencies were observed based on age, vascular invasion, or M classification (Fig. 9B, D, and G).

## Discussion

CRC is a highly heterogeneous disease caused by the interaction between genetic and environmental factors. Multiple alternative genetic pathways exist in CRC development. CRC evolves from benign to malignant lesions, and many mutations in oncogenes and tumor suppressor genes are involved in CRC tumorigenesis and progression [30, 31]. CRC characterized by high mutation levels is thought to harbor an increased neoantigen burden, which is highly immunogenic and sensitive to immune checkpoint inhibitor (ICI) therapy [32]. The investigation of predictive and prognostic biomarkers has historically focused on alterations in the RAS/BRAF/MEK/MAPK and PI3K/AKT/mTOR pathways. For the RAS/BRAF/MEK/MAPK pathway, fruitful and solid evidence has emerged supporting the use of BRAF as a prognostic biomarker and RAS as a predictive biomarker in CRC [33]. However, biomarkers associated with the PI3K/AKT/mTOR pathway, such as PIK3CA and PTEN, are not recommended for use in clinical practice due to insufficient evidence, especially associated with CRC treatment [34, 35]. Therefore, identifying other components of the PI3K/AKT/mTOR pathway that may serve as prognostic biomarkers for targeted therapy and ICI remains necessary. In this study, we focused on mTOR, which is a master regulator of the PI3K/AKT signaling pathway. The role of MTOR in CRC has been under-characterized, and we aimed to dissect its biological functions and reveal its associations with responses to targeted therapies.

The pan-cancer analysis showed that *MTOR* mRNA is overexpressed in many cancers. However, analysis of RNA sequencing data from TCGA (COAD and READ) suggested no significant differences in mTOR expression between tumor tissues and normal tissues, which was inconsistent with previous findings [20–22]. Considering the potential for ethnic differences to affect tumorigenesis, we further selected expression data associated with CRC among an Asian population from the GEO database for analysis and identified that the expression of *MTOR* in tumor tissues was upregulated compared with normal tissues in an ethnicity-dependent manner in an Asian cohort.

To analyze the prognostic value of *MTOR* among CRC patients, we divided CRC patients from the TCGA dataset into high and low *MTOR* expression groups based on the median and quartile levels. No significant difference was expected between groups based on the median level because the expression of *MTOR* in cancer tissues did not differ significantly from that in normal tissues. However, when analyzed according to the quartile level (top 25% and bottom 25%), the high *MTOR* expression group was significantly associated with poor survival, which suggested that differences in *MTOR* expression level were associated with prognosis. A similar trend was also found among CRC patients from the GEO database, although the differences in the GEO dataset did not reach significance, possibly due to the small sample size. These results indicated the potential for *MTOR* to serve as a prognostic marker in Asian CRC patients, although this possibility requires further clinical validation.

Pathway analysis and GSEA results showed that DEGs were enriched in the metabolism, cell adhesion, and translation pathways. *MTOR* was associated with a key fatty acid, glutathione, and oxidative phosphorylation metabolic pathways in CRC. Fatty acids are indispensable for the synthesis of membranes and signaling molecules, which are associated with cell proliferation [36]. As an antioxidant, glutathione has profound effects on cell survival while also conferring therapeutic resistance to cancer cells [37]. Oxidative phosphorylation is a primary source of energy. Studies have shown that mitochondrial DNA (mtDNA) content is higher in CRC than in normal tissue, which may indicate a higher contribution of oxidative phosphorylation in CRC [38]. *MTOR* serves as a key regulator of these metabolic pathways [39–41]. In addition, *MTOR* regulates protein translation and synthesis to promote cell proliferation. The proliferation of CRC cells can be disrupted by reducing the phosphorylation level of eIF4E-binding protein 1 (4EBP1), which is a tumor suppressor protein activated by mTOR [42]. Cell adhesion is a key mediator of cancer progression and facilitates several behavioral hallmarks of cancer, including immune evasion and metastatic dissemination [43]. Further study found that mTOR complex (mTORC)1 and mTORC2 were both involved in the regulation of cell adhesion [44]. Therefore, *MTOR* might affect these signaling pathways, contributing to a poor prognosis in CRC patients.

Our study revealed that *MTOR* expression was associated with a high somatic mutation burden. In theory, tumors with a higher number of genetic variations are statistically more likely to generate novel mutant proteins or neoantigens, which may be recognized as foreign invaders by the immune system and trigger a cytotoxic, tumor-killing response [45]. Relevant analyses revealed a correlation between TMB and the response rates and outcomes of ICI therapy [46]. ICI therapy has shown promising results in various types of cancers, particularly antibodies against the programmed cell death protein 1 (PD-1) T-cell coreceptor and its ligand B7-H1/programmed death-ligand 1 (PD-L1), which have induced durable tumor responses, even in late-stage patients who have failed to respond to multiple classical treatment strategies [47, 48]. Previous studies have indicated that immune infiltration levels are related to prognosis and the response to ICI therapy for several cancers, including esophageal squamous cell carcinoma, breast cancer, and CRC [49–51]. In the TME, we found that the *MTOR* expression and CNAs significantly affected the immune cell infiltration of CD8^+^ T cells, B cells, neutrophils, and dendritic cells in CRC. Using CIBERSORT, our results showed that CD8^+^ T cells were negatively correlated with *MTOR* expression. In addition, we found that the expression levels of *PDCD-1*, *CTLA4*, and *LAG3* were positively associated with *MTOR* expression. These results indicated that *MTOR* status could potentially be used to assess whether patients might benefit from ICI therapy.

Moreover, our results showed that the expression and function of *MTOR* were altered in MSI-H CRC patients. MSI is an intensively studied biomarker with prognostic and therapeutic values in CRC. MSI refers to changes in the lengths of short-tandem-repeat DNA sequences, the presence of which represents phenotypic evidence of deficient mismatch repair (dMMR). MMR is a highly conserved cellular process intended to correct erroneous insertions, deletions, and base–base mismatches that occur during DNA replication and recombination and have escaped the proofreading process. When the MMR system develops a malfunction, errors generated during DNA replication increase, including single-base substitutions, insertions, or deletions of short-tandem-repeat DNA sequences, resulting in MSI [52]. MSI is present in approximately 15% of CRC and 5% of metastatic CRC (mCRC) [53, 54]. Depending on the degree of instability, MSI tumors can be divided into MSI-H or MSI-L subsets. Several clinical studies have found that patients with MSI-H present with a durable and robust response to ICI therapy [55, 56]. Although the response rates to ICI among patients with MSI-H CRC have been variable in different trials, more somatic mutations and higher neoantigen burdens were identified in responsive tumors than in non-responsive tumors [57].

Few studies have reported the correlation between *MTOR* and MSI-H. Vilar et al. [58] reported that MSI-H cell lines responded better to therapies that preferentially target the PI3K/AKT/mTOR pathway but did not explore the expression of *MTOR* in MSI-H. Consistent with these results, we revealed that rapamycin preferentially targeted MSI-H cell lines via cytotoxicity experiments. Additionally, we found that the expression of *MTOR* increased in MSI-H CRC samples from a public database, which was then validated in both cell lines and CRC cohorts from our center. Lin et al. reported that MSI-H CRC tumors feature a significantly increased number of mTOR pathway mutations than MSS tumors [59]. In addition, evidence has shown that *MTOR* mutants can constitutively activate the mTOR signaling pathway and increase the sensitivity to rapalog treatment [60]. Therefore, whole-exome sequencing in CRC patients (MSI-H or MSS) was applied to detect variations among all SNP sites in *MTOR*. To the best of our knowledge, this is the first systematic study examining the relationship between *MTOR* mutations and CRC. Our analysis showed that the mutation frequency of *MTOR* in MSI-H patients was significantly increased compared with MSS patients, which can affect the protein activity of mTOR and influence the tumor response to rapamycin treatment. Furthermore, we observed a significant increase in the frequency of transition mutations in the MSI-H group compared with that in the MSS group, which may be attributed to dMMR. Studies on MMR function have shown that MMR has a higher repair efficiency for transition mutations than other mutations [61, 62]. These results suggested that *MTOR* mutations may present at a higher frequency in CRC patients with dMMR. Combined with the relationship between *MTOR* and high TMB, MTOR status could potentially be used to assess whether MSI-H patients might benefit from ICI therapy. Further research examining the efficacy of ICI therapy in *MTOR*-mutant MSI-H CRC remains necessary.

In this study, we found that *MTOR* played an important role in tumorigenesis and was associated with the immunological status of CRC. The function of mTOR in immunity has been extensively studied. Several studies have reported that the mTOR signaling pathway affects the function of immune cells and cytokines in the TME [63–65]. In our study, *MTOR* was shown to be associated with the infiltration of various immune cells. Moreover, our study found that the high expression of *MTOR* was related to high TMB. Tumors with higher numbers of genetic variations are more likely to generate novel mutant proteins or neoantigens. Therefore, *MTOR* is likely not only associated with immune cell function but also with tumor cell immunogenicity, suggesting that *MTOR* may play a central role in tumor immunity. Interestingly, we found that *MTOR* was associated with MSI status, which was characterized by high TMB and abundant TIICs. The consistent relationship identified between *MTOR* mutations and MSI suggests that *MTOR* may represent a marker for the prediction of MSI status and tumor immunogenicity. Yang et al. [66] reported that MSI CRC tumors had higher expression levels of thymocyte selection–associated high-mobility group box (TOX), an inhibitor of mTOR, compared with MSS tumors. Our research focused on *MTOR* mutations rather than *MTOR* expression levels, and the work by Yang et al. did not examine the relationship between TOX expression and *MTOR* mutation.

The significant contributions of *MTOR* to CRC tumorigenesis suggest that mTOR inhibitors may be effective for CRC therapy. Several clinical trials have studied the efficacy of mTOR inhibitors in CRC patients (A supplementary table shows this in more detail). For example, in a clinical phase I/II study, everolimus combined with mFOLFOX-6 and bevacizumab was found to be tolerable and demonstrated preliminary efficacy for mCRC therapy. The objective response rate was 53% in mCRC patients and was higher (86%) in those cases with PTEN deficiency [67]. Studies on other types of inhibitors, such as ATP-competitive mTOR inhibitors and mTOR/PI3K dual inhibitors, have also shown tumor growth inhibition effects against CRC cell lines and xenograft models [68, 69]. Moreover, studies have found that BEZ235, an mTOR/PI3K dual inhibitor, was capable of inducing a treatment response and overcoming resistance to everolimus in *APC*- and *PIK3CA*-mutant CRC cells [70, 71]. These findings suggested that different molecular subtypes might be associated with mTOR inhibition responses, which could be used to distinguish patients who will benefit from mTOR inhibition therapy. Further research examining the efficacy of mTOR inhibitors in *MTOR*-mutant remains necessary.

Moreover, the status of *MTOR* has been shown to regulate immunoreactions. Rapamycin is widely used as an immunosuppressant to prevent immune rejection in kidney transplant patients. Jung et al. found that rapamycin uniquely enhanced the number and function of CD8^+^ effector and central memory T cells [72]. In a mouse model of RCC, anti-PD-L1 combined with everolimus was more effective for tumor regression than individual treatment due to the upregulation of PD-L1 in tumor cells, which increased the tumor-infiltrating CD8^+^ T cells [73]. Similarly, our study demonstrated that the status of *MTOR* could be used to assess the immunological function of CRC patients and might serve as a potential indicator that can predict the optimum response to ICI therapy. mTOR inhibitors that promote cancer cell death and boost effector functions in T cells can be combined to improve ICI therapeutic outcomes. The relationship between *MTOR* mutations and dMMR suggests that CRC patients with dMMR are likely to benefit from combination therapy. It is worth noting that several studies have found the efficacy of rapamycin is likely to be based on dosage [74]. Like a double-edged sword, too low-dose rapamycin was insufficient to activate its therapeutic effects on the diseases, such as mitochondrial disease [75]. However, too high-dose rapamycin is clinically toxic. Low-dose rapamycin could enhance the quantity and quality of CD8^+^T cells, while the higher doses of rapamycin inhibited the T-cells response [76]. Therefore, it is meaningful and could be challenging, to further explore the most appropriate dose of rapamycin to strike a balance between stimulating immunity and anti-tumor activity.

In summary, our study found that *MTOR* plays an important role in CRC tumorigenesis and was associated with prognosis, metabolism, and immune. Furthermore, adenosine monophosphate-activated protein kinase (AMPK) has been found to be involved in regulating mTOR and mTOR regulated pathways in a feedback loop manner [77, 78]. 5-Aminoimidazole-4-carboxamide-1-β-4-ribofuranoside, an AMPK activator, could inhibit mTORC1 and promote the therapeutic effect of rapamycin in cancers [79]. The similarity of compounds to rapamycin in transcriptional signature have been previously shown to have rapamycin-like properties [80]. Therefore, elaborating this connection will be a future aspect of our study, providing new light on the role of AMPK and AMPK activators in CRC.

## Conclusion

In conclusion, our study found that *MTOR* plays an important role in CRC tumorigenesis and was associated with prognosis, TMB, TIICs, and MSI status. Therefore, *MTOR* may represent a comprehensive indicator of prognosis and immunological status for CRC. Moreover, the mutation profile of *MTOR* may provide guidance for subsequent clinical research on mTOR inhibitors in CRC.

## Supplementary Information

**Additional file 1: Supplementary fig. 1. A- B** TMB of the low and high *MTOR* expression groups was compared with different cancer cohorts in TCGA. Every dot represents a sample, and the red horizontal lines represent the median numbers of mutations in the respective cancer types. **C- D** Differences in the proportions of affected pathways between the low and high *MTOR* expression groups. TMB, tumor mutational burden; TCGA, The Cancer Genome Atlas.

**Additional file 2: Supplementary fig. 2.** The inhibition curve showing the differential sensitivity of CRC cell lines to treatment with rapamycin. CRC, colorectal cancer.

**Additional file 3: Supplementary fig. 3.** This is a full-length blot of mTOR, and the labeled portion was used for Fig.7.

**Additional file 4: Supplementary fig. 4.** This is a full-length blot of p-mTOR, and the labeled portion was used for Fig.7.

**Additional file 5: Supplementary fig. 5.** This is a full-length blot of GAPDH, and the labeled portion was used for Fig.7.

**Additional file 6: Supplementary Table.** Clinical trials investigating mTOR inhibitors in CRC.

## Data Availability

The raw sequence data reported in this paper have been deposited in the Genome Sequence Archive [81] in National Genomics Data Center [82], China National Center for Bioinformation / Beijing Institute of Genomics, Chinese Academy of Sciences, under accession number HRA000775 that are publicly accessible at https://bigd.big.ac.cn/gsa.

## Abbreviations

mTOR/*MTOR*: Mammalian target of rapamycin
PI3K: Phosphoinositide 3-kinase
AKT: Protein kinase B
RCC: Renal cell carcinoma
CRC: Colorectal cancer
PTEN: Phosphatase and tensin homolog
PIK3CA: Phosphatidylinositol-4,5-bisphosphate 3-kinase catalytic subunit alpha
TMB: Tumor mutational burden
MSI: Microsatellite instability
MSI-H: MSI-high
MSI-L: MSI-low
MSS: Microsatellite stable
TCGA: The Cancer Genome Atlas
GEO: Gene Expression Omnibus
GO: Gene Ontology
KEGG: Kyoto Encyclopedia of Genes and Genomes
GSEA: Gene set enrichment analysis
COAD: Colon carcinoma
READ: Rectal carcinoma
TIMER: Tumor Immune Estimation Resource
DEGs: Differentially expressed genes
TME: Tumor microenvironment
IC_50_: Half maximal inhibitory concentration
TIICs: Tumor-infiltrating immune cells
CNAs: Copy number alterations
SNP: Single-nucleotide polymorphisms
ICI: Immune checkpoint inhibitor
AMPK: Adenosine monophosphate-activated protein kinase
