# Supplementary material for: Genomic signature of MTOR could be an immunogenicity marker in human colorectal cancer
Source: BMC Cancer. 2022 Jul 26;22:818. doi: 10.1186/s12885-022-09901-w (PMC9327395; doi:10.1186/s12885-022-09901-w)
Supplement: Supplementary file 1 — Additional file 1: Supplementary fig. 1. A- B TMB of the low and high MTOR expression groups was compared with different cancer cohorts in TCGA. Every dot represents a sample, and the red horizontal lines represent the median numbers of mutations in the respective cancer types. C- D Differences in the proportions of affected pathways between the low and high MTOR expression groups. TMB, tumor mutational burden; TCGA, The Cancer Genome Atlas. [file 12885_2022_9901_MOESM1_ESM.pdf]

Supplementary Fig.1

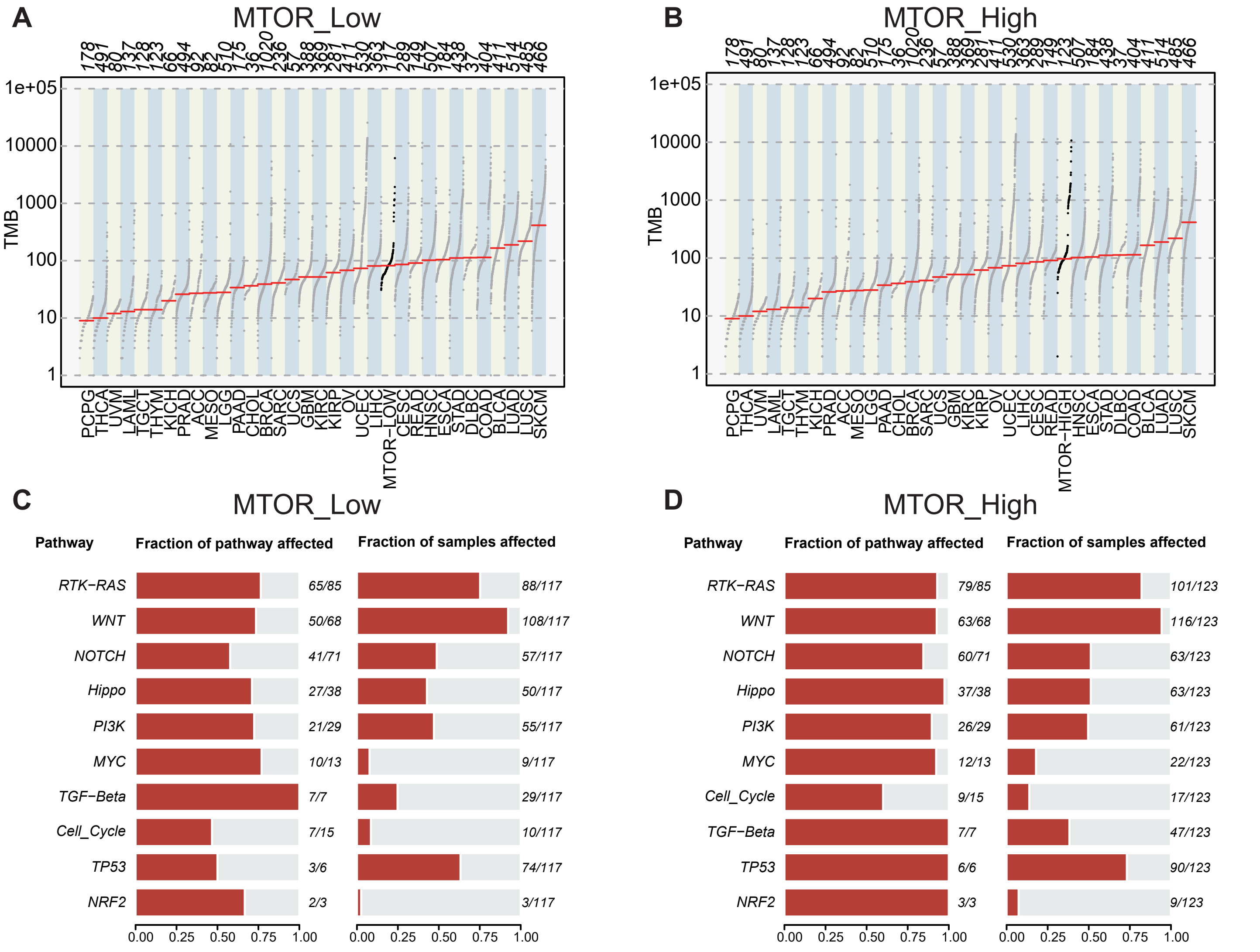

A-B TMB of the low and high *MTOR* expression groups was compared with different cancer cohorts in TCGA. Every dot represents a sample, and the red horizontal lines represent the median numbers of mutations in the respective cancer types. **C-D** Differences in the proportions of affected pathways between the low and high *MTOR* expression groups. TMB, tumor mutational burden; TCGA, The Cancer Genome Atlas.
