# Supplementary material for: Genomic signature of MTOR could be an immunogenicity marker in human colorectal cancer
Source: BMC Cancer. 2022 Jul 26;22:818. doi: 10.1186/s12885-022-09901-w (PMC9327395; doi:10.1186/s12885-022-09901-w)
Supplement: Supplementary file 2 — Additional file 2: Supplementary fig. 2. The inhibition curve showing the differential sensitivity of CRC cell lines to treatment with rapamycin. CRC, colorectal cancer. [file 12885_2022_9901_MOESM2_ESM.pdf]

Supplementary Fig.2

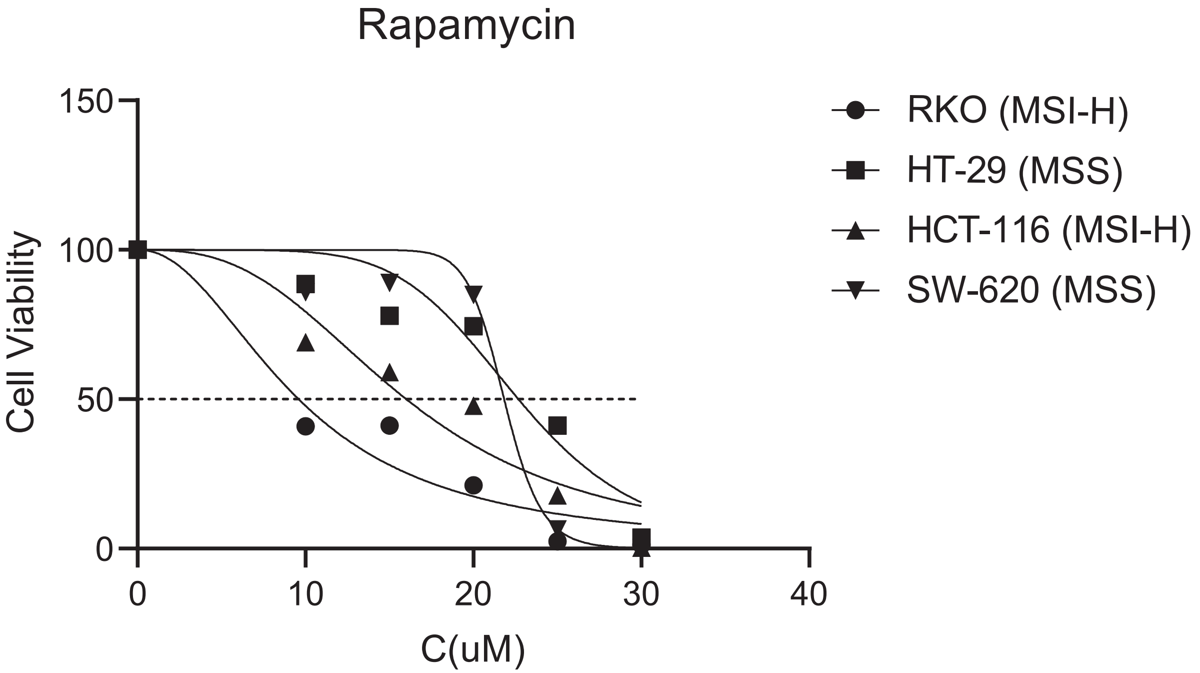

The inhibition curve showing the differential sensitivity of CRC cell lines to treatment with rapamycin. CRC, colorectal cancer.
