# Supplementary figures and images for: Genomic signature of MTOR could be an immunogenicity marker in human colorectal cancer
Source: BMC Cancer. 2022 Jul 26;22:818. doi: 10.1186/s12885-022-09901-w (PMC9327395; doi:10.1186/s12885-022-09901-w)

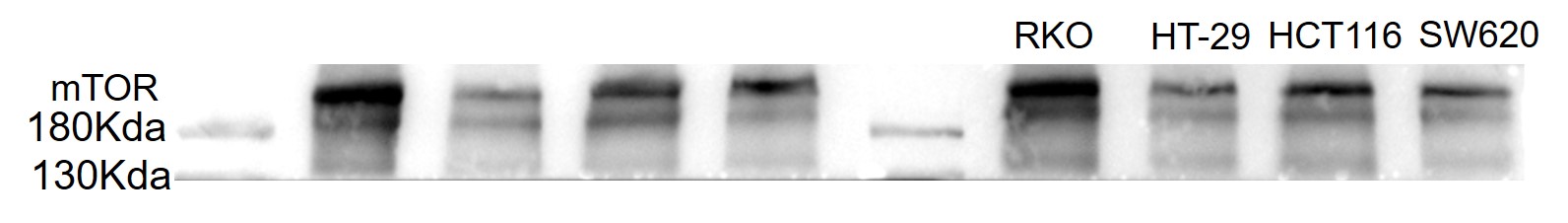

Supplement: Supplementary file 3 — Additional file 3: Supplementary fig. 3. This is a full-length blot of mTOR, and the labeled portion was used for Fig.7. [file 12885_2022_9901_MOESM3_ESM.jpg]

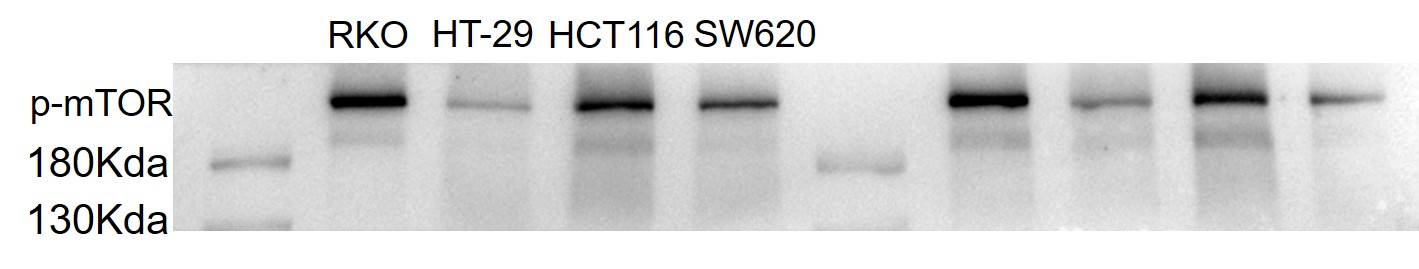

Supplement: Supplementary file 4 — Additional file 4: Supplementary fig. 4. This is a full-length blot of p-mTOR, and the labeled portion was used for Fig.7. [file 12885_2022_9901_MOESM4_ESM.jpg]

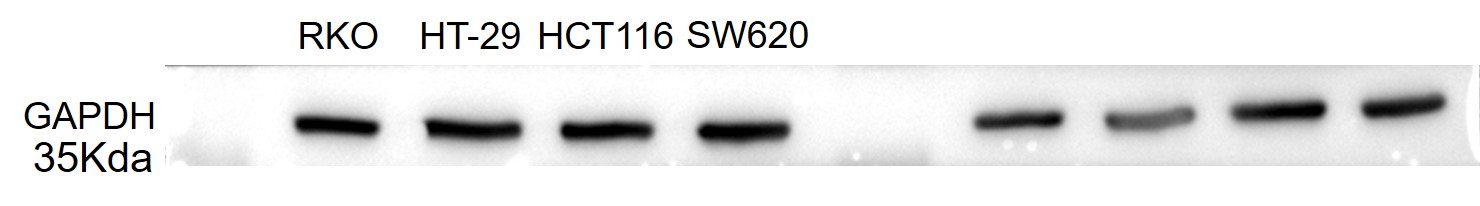

Supplement: Supplementary file 5 — Additional file 5: Supplementary fig. 5. This is a full-length blot of GAPDH, and the labeled portion was used for Fig.7. [file 12885_2022_9901_MOESM5_ESM.jpg]
