# Supplementary material for: Genomic signature of MTOR could be an immunogenicity marker in human colorectal cancer
Source: BMC Cancer. 2022 Jul 26;22:818. doi: 10.1186/s12885-022-09901-w (PMC9327395; doi:10.1186/s12885-022-09901-w)
Supplement: Supplementary file 6 — Additional file 6: Supplementary Table. Clinical trials investigating mTOR inhibitors in CRC. [file 12885_2022_9901_MOESM6_ESM.docx]

**Supplementary Table** Clinical trials investigating mTOR inhibitors in CRC.

| **Drug(s)** | **Clinical Trial Number** | **Phase** | **Primary Endpoint(s)** |
| --- | --- | --- | --- |
| Everolimus | NCT00419159 | 2 | Disease Control Rate  Objective Response Rate  The Number of Participants with Best Overall Response |
| Everolimus and OSI-906 | NCT01154335 | 1 | Maximum Tolerated Dose |
| Everolimus and Tivozanib | NCT01058655 | 1/2 | Maximum Tolerated Dose  Dose Limiting Toxicity  Progression-Free Survival |
| Everolimus and PDR001 | NCT02890069 | 1 | Dose Limiting Toxicity |
| Everolimus with FOLFOX and Bevacizumab | NCT01047293 | 1 | Dose Limiting Toxicity  Progression-Free Survival |
| ABI-009 with FOLFOX and Bevacizumab | NCT03439462 | 1/2 | Incidence of Treatment-Emergent Adverse Events  Progression-Free Survival |
| ABI-009 and Nivolumab | NCT03190174 | 1/2 | Maximum Tolerated Dose |
| SAR245409 and pimasertib | NCT01390818 | 1 | Dose Limiting Toxicity |
